# Correlation profiling of brain sub-cellular proteomes reveals co-assembly of synaptic proteins and subcellular distribution

**DOI:** 10.1038/s41598-017-11690-3

**Published:** 2017-09-21

**Authors:** Nikhil J. Pandya, Frank Koopmans, Johan A. Slotman, Iryna Paliukhovich, Adriaan B. Houtsmuller, August B. Smit, Ka Wan Li

**Affiliations:** 1 Department of Molecular and Cellular Neurobiology, Center for Neurogenomics and Cognitive Research, Amsterdam Neuroscience, Vrije Universiteit Amsterdam, Amsterdam, The Netherlands; 2 Department of Functional Genomics, Center for Neurogenomics and Cognitive Research, Amsterdam Neuroscience, Vrije Universiteit Amsterdam, Amsterdam, The Netherlands; 3 Optical Imaging Center, Department of Pathology, Erasmus Medical Center, 3015 GE Rotterdam, Netherlands

## Abstract

Protein correlation profiling might assist in defining co-assembled proteins and subcellular distribution. Here, we quantified the proteomes of five biochemically isolated mouse brain cellular sub-fractions, with emphasis on synaptic compartments, from three brain regions, hippocampus, cortex and cerebellum. We demonstrated the expected co-fractionation of canonical synaptic proteins belonging to the same functional groups. The enrichment profiles also suggested the presence of many novel pre- and post-synaptic proteins. Using super-resolution microscopy on primary neuronal culture we confirmed the postsynaptic localization of *PLEKHA5* and *ADGRA1*. We further detected profound brain region specific differences in the extent of enrichment for some functionally associated proteins. This is exemplified by different AMPA receptor subunits and substantial differences in sub-fraction distribution of their potential interactors, which implicated the differences of AMPA receptor complex compositions. This resource aids the identification of proteins partners and subcellular distribution of synaptic proteins.

## Introduction

Brain function is carried in large by synaptic transmission between neurons. Synaptic transmission relies on the stimulus-dependent release of transmitters from the presynaptic element and receptor-mediated signal perception and integration at the postsynapse. In both elements of the synapse crucial functional assemblies of proteins have been identified underlying synaptic function. These include the interaction of t- and v-SNARE proteins *STX1/2*, *SNAP25* and *VAMP2* ^1–4^, facilitating the Ca^2+^ influx-induced fusion of docked/primed synaptic vesicles to the membrane of the presynaptic active zone. This event leads to the exocytotic release of glutamate which binds to and activates NMDA- and AMPA-type receptors residing in the opposing post-synaptic membrane^5^. These receptors in turn are anchored to the postsynaptic density (PSD) via an assembly of scaffolding proteins belonging, among others, to *DLG* and *SHANK* families^6^.

Using proteomics analysis various molecular machineries governing synaptic sub-function, have been characterized successfully, including the synaptic vesicle, the pre-synaptic active zone and PSD^7–14^. In most cases these topological synaptic subdomains were isolated biochemically, and subsequently subjected to proteomics analysis to define its constituents. A caveat of this is that although the sample is enriched in proteins from the targeted compartment it contains proteins from other compartments, albeit at a lower level. Correlation profiling^15^ (also known as fractionation/spatial profiling^16,17^) of the step-wise isolated fractions is a preferred method to deal with protein enrichment in fractions, from which their co-assembly or subcellular localization might be inferred.

Here we applied data-dependent mass spectrometry on five biochemically isolated cellular sub-fractions, with focus on synaptic compartments, from three mouse brain regions, hippocampus, cortex and cerebellum. In each brain region at least 3000 proteins were quantified with a total of 4237 unique proteins in the complete dataset, with a sub-fraction typically yielding ~2000 proteins. Correlation profiling of some well-known synaptic functional groups showed enrichment of pre-synaptic proteins in synaptosome or canonical postsynaptic proteins in the PSD.

The AMPA receptor (AMPAR), a genuine postsynaptic receptor, showed considerable brain region-specific distribution profiles. Also, the recently reported high-confident AMPAR interacting proteins^18–20^ followed different distribution patterns. These data suggest the presence of spatially segregated, distinct AMPAR sub-complexes. Our analyses further suggested the presence of novel synaptic proteins. We validated PSD localization of *PLEKHA5* and *ADGRA1* by super-resolution microscopy. Together, the present study provides a rich resource to interrogate the potential co-assembly and subcellular distribution of synaptic proteins in hippocampus, cortex, and cerebellum.

## Results and Discussion

### Generating a synaptic proteome resource for correlation profiling

**Figure 1:**
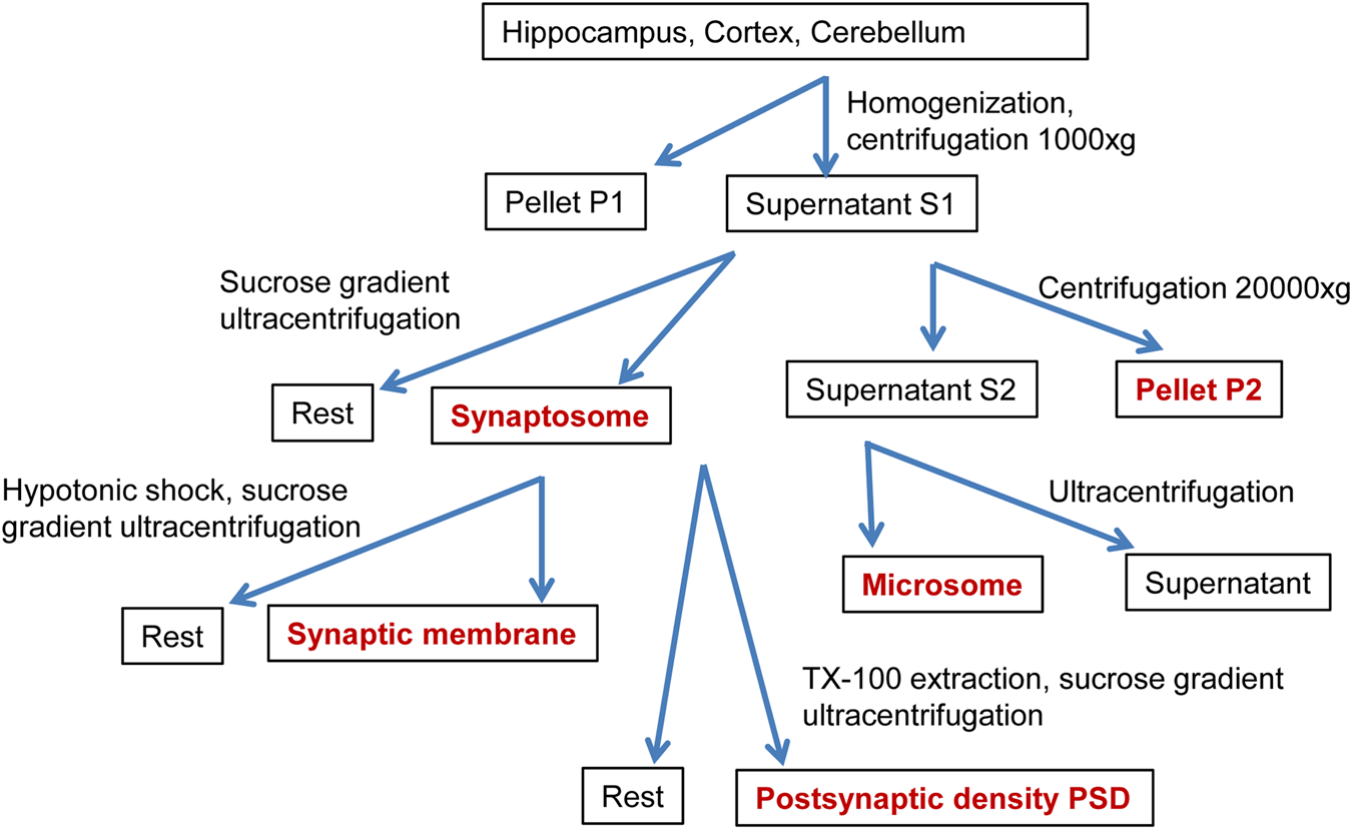
Biochemical isolation of cellular sub-fractions from three brain regions. Fractions labelled red were collected for proteomics analysis.

We isolated biochemical cellular sub-fractions, with emphasis on synaptic compartments, from three brain regions (Fig. 1) followed by label-free data dependent MS analysis of each fraction similar to previously described spatial/fractionation correlation proteomics^16^. Data visualization methods were chosen to emphasize the (1) quantitative fraction distribution of a few selected proteins by their iBAQ values, or the (2) normalized fraction distribution of a large number of proteins to accommodate the profound quantitative differences that span 4 orders of magnitude. First, the experimental reproducibility was addressed. Biological replicates of proteomics analyses were performed on hippocampal microsome (M), P2, synaptosome (SYN), synaptic membrane (SYM) and postsynaptic density (PSD). Three replicates of P2 and synaptic membrane, show each R^2^ of at least 0.856, which is representative of all synapse sub-fractions (Supplemental Fig. S1). For PSD we have two replicates with similar reproducibility. Overall, the coefficient of variation of each synaptic fraction ranges from 26–34% (Supplemental Fig. S1), as is typical for data-dependent analysis, and has been reported previously for other biological systems^21^.

**Figure 2:**
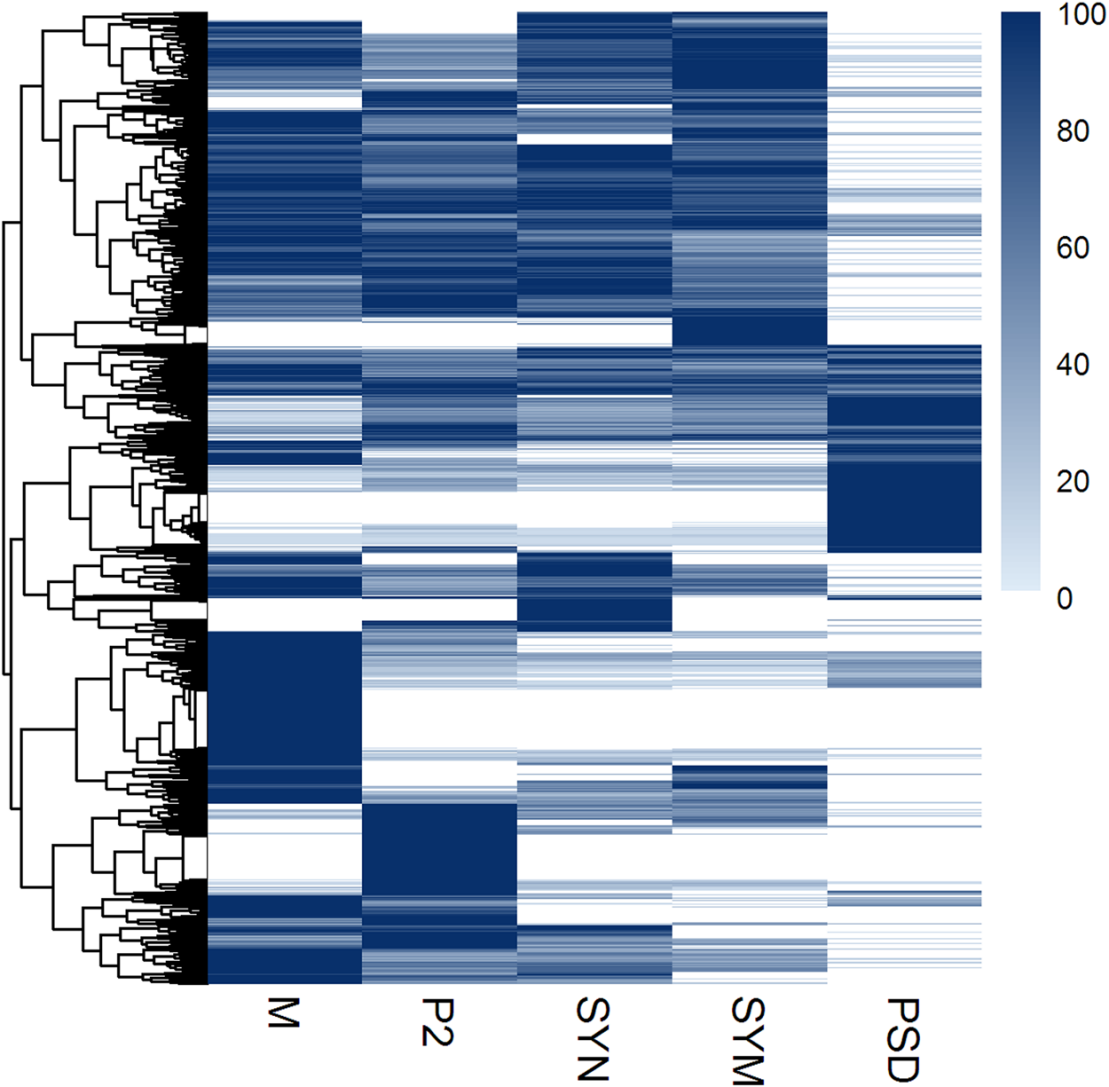
Hierarchical clustering of 3632 proteins shows specific enrichment in various subcellular fractions of the hippocampus. Protein mean abundances were scaled between zero and 100% of their maximum over all sub-fractions.

Approximately 2000 proteins were identified in each sub-fraction, with the exception of PSD in which about 1400 identified proteins were detected, likely reflecting lower sample complexity. The dynamic range of the protein abundances in each fraction spans over four orders of magnitude (Supplemental Fig. S1). To reveal the distribution of each protein across the synapse sub-fractions within a brain region, we scaled protein abundances between zero and 100% of their maximum value over all sub-fractions. Hierarchical cluster analysis of 3632 proteins quantified in five hippocampus sub-fractions revealed the presence of sub-fraction-specific groups of proteins (Fig. 2). Only 166 proteins, ~6% of all proteins quantified in the hippocampus sub-fractions, were found exclusively in P2, whereas the remaining proteins showed a variety of more complex enrichment profiles for the other sub-fractions. The differential distribution of proteins across the fractions was at the basis for subsequent correlation profiling analysis.

**Figure 3:**
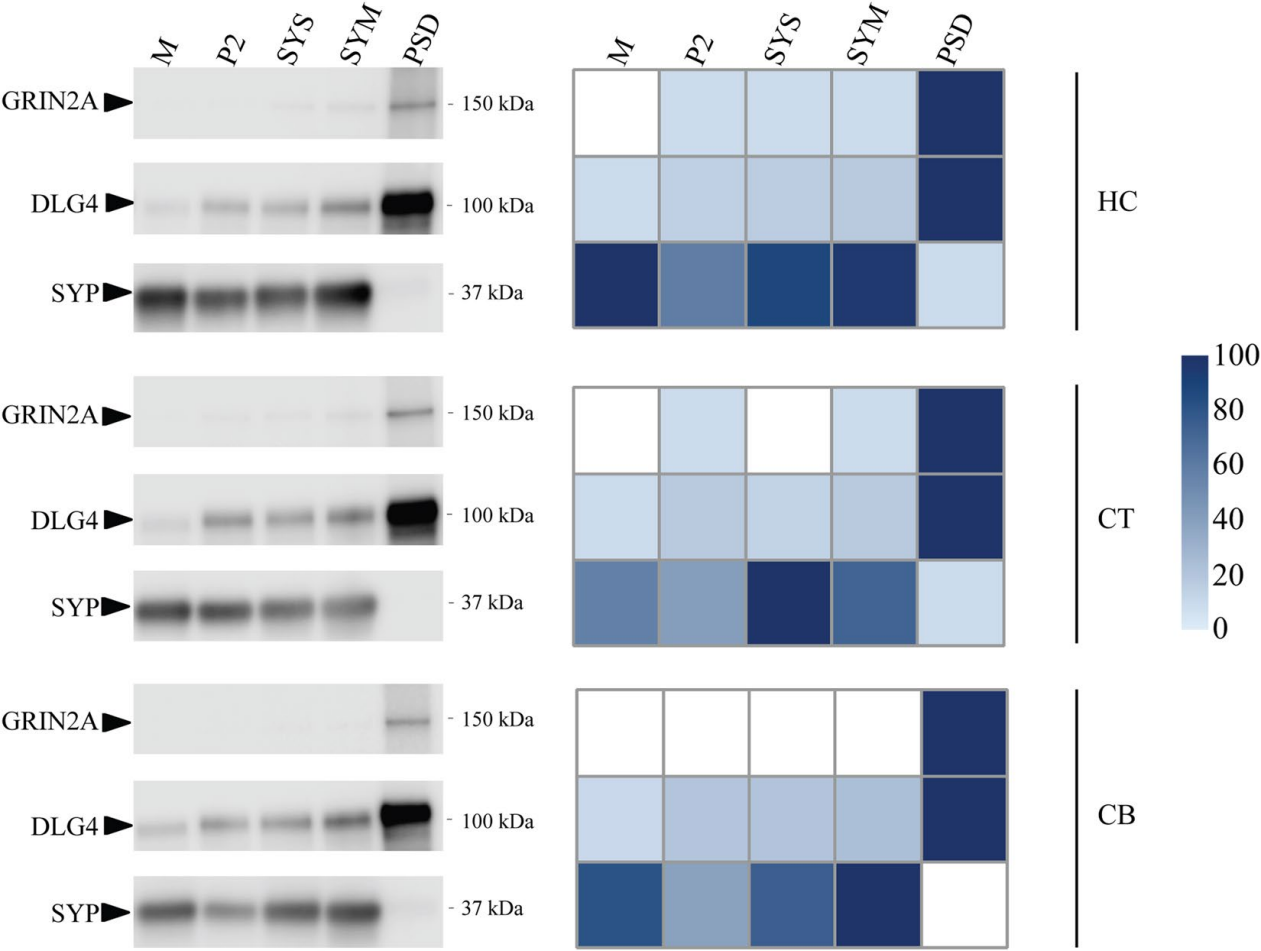
Protein abundance distribution over the cellular sub-fractions in hippocampus (HC), cortex (CT) and cerebellum (CB). Left panel is an immunoblot analysis showing the presence of presynaptic protein *SYP*, and postsynaptic proteins *GRIN2A* and *DLG4* in each sub-fraction. Right panel shows protein abundance in each sub-fraction as observed with proteomics. Mass spectrometry protein abundance values were scaled between zero and 100% of their maximum over all sub-fractions in each panel and accordingly color-coded from light-blue to dark-blue.

Next, we carried out the same analysis of cortical and cerebellar synapse sub-fractions and show the protein abundance matrices for all the fractions from cortex, cerebellum and hippocampus (Supplemental Table S1). As the analysis of hippocampal samples revealed a low variation of biological replications, the analysis of single samples of cortex and cerebellum should be sufficient. Hierarchical cluster analyses of proteins quantified in the cellular sub-fractions from cortex and cerebellum are shown in Supplementary Fig. S2. Immuno-blotting was performed on a selection of canonical pre- and postsynaptic proteins, the presynaptic vesicle protein *SYP* and the postsynaptic NMDA receptor subunit *GRIN2A*, and the scaffolding protein *DLG4* (PSD95/SAP90) that anchors NMDA receptor and other proteins to the PSD, to validate the data as revealed by mass spectrometry. The similarity of protein distribution profiles between these two different measurement methods is high (Fig. 3). *SYP* was found enriched in synaptosome and synaptic membrane and highly depleted or absent in the PSD fraction. *GRIN2A* and *DLG4* were highly enriched in PSD. Thus, immunoblotting confirmed the mass spectrometry based data.

### Functional grouping by correlation profiling

**Figure 4:**
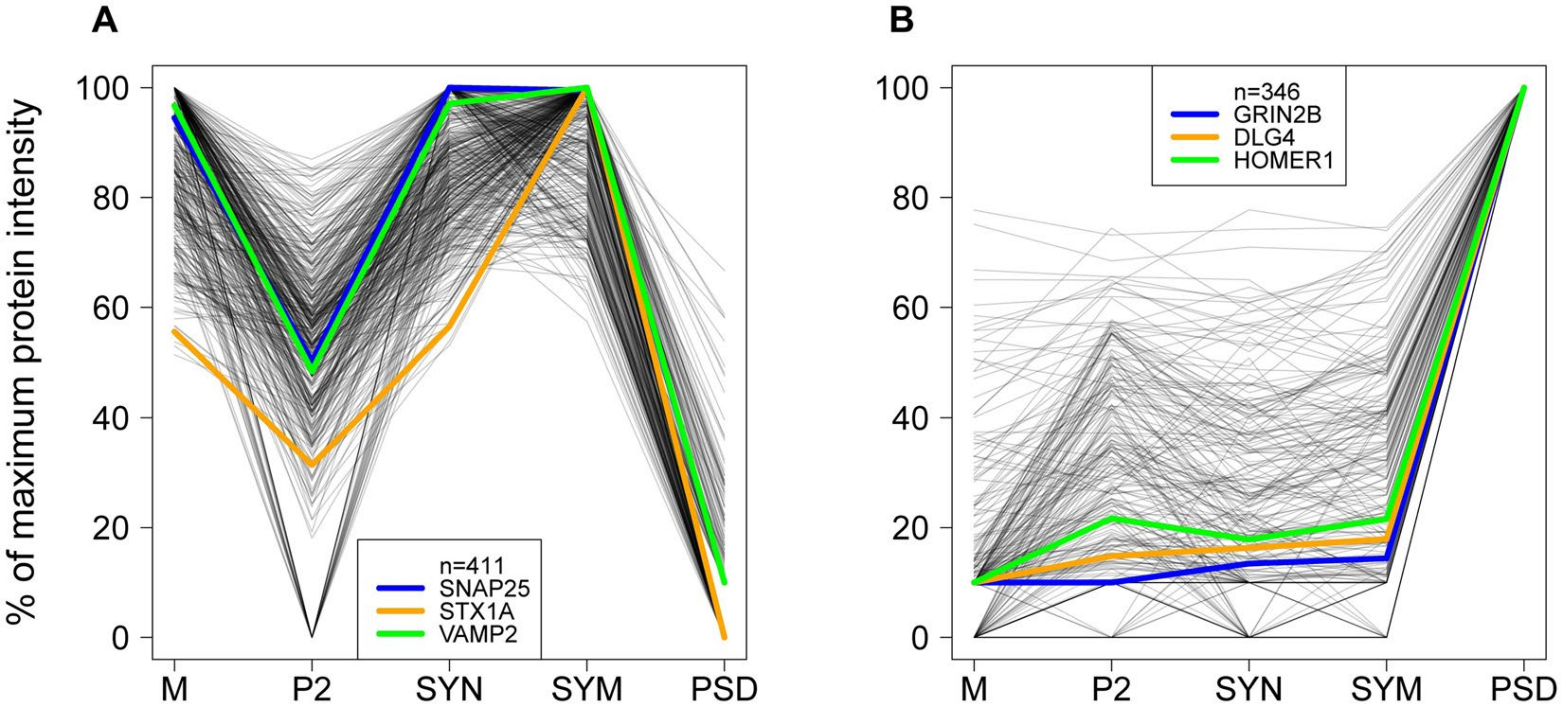
Correlation analysis of abundance profiles of proteins over sub-fractions of the hippocampus with selected seed proteins. In each panel, three functionally related seed proteins were chosen typical for each of the specific synaptic processes in (**A**) and (**B**). Proteins shown have a Pearson correlation of at least 0.9 with at least two out of these three seed proteins. Protein abundances are scaled between zero and their maximum intensity over all selected sub-fractions. This leads to A) exocytosis, 411 proteins and B) postsynaptic density, 346 proteins.

We next investigated whether proteins might be delineated that assemble into correlated functional groups and are related to known synaptic processes. For this we used the protein abundance profiles over sub-fractions of the hippocampus. Correlation profiling was performed using three ‘seeds’ that are well-known representatives of SV exocytosis and the PSD, respectively. Protein profiles in the entire catalog, with a Pearson correlation of ≥0.9 with two of the three seed proteins, were selected (Fig. 4, Supplemental Table S2). Proteins strongly correlated with the exocytosis seeds were enriched in synaptosomes, synaptic membranes and the microsomal fraction and were found depleted in P2 and PSD (Fig. 4A). Conversely, the seed proteins for the PSD group were depleted in synaptosomes, synaptic membranes and microsomes (Fig. 4B), but with a larger variation in their enrichment for PSD compared to other fractions. Proteins of the PSD are in many cases differentially detected in other fractions, potentially indicative of differences in assembly during routing to the PSD, or alternatively, being part of different functional units within and outside the PSD. Obviously, the inclusion criteria for similar profiles can be adjusted. For example, the correlation coefficient threshold might be adjusted, or one might opt for a more stringent setting with inclusion of more seed proteins. The impact on the number of correlated proteins and trade-off in true/false-positives can be explored with provided data based on user-specified criteria (see example Supplemental Fig. S3 and Supplemental Table S2). The correlation profiling of each protein against all other identified proteins in hippocampus, cortex and cerebellum, are shown in Supplementary Tables S3–5, respectively.

### Subcellular localization by correlation profiling

**Figure 5:**
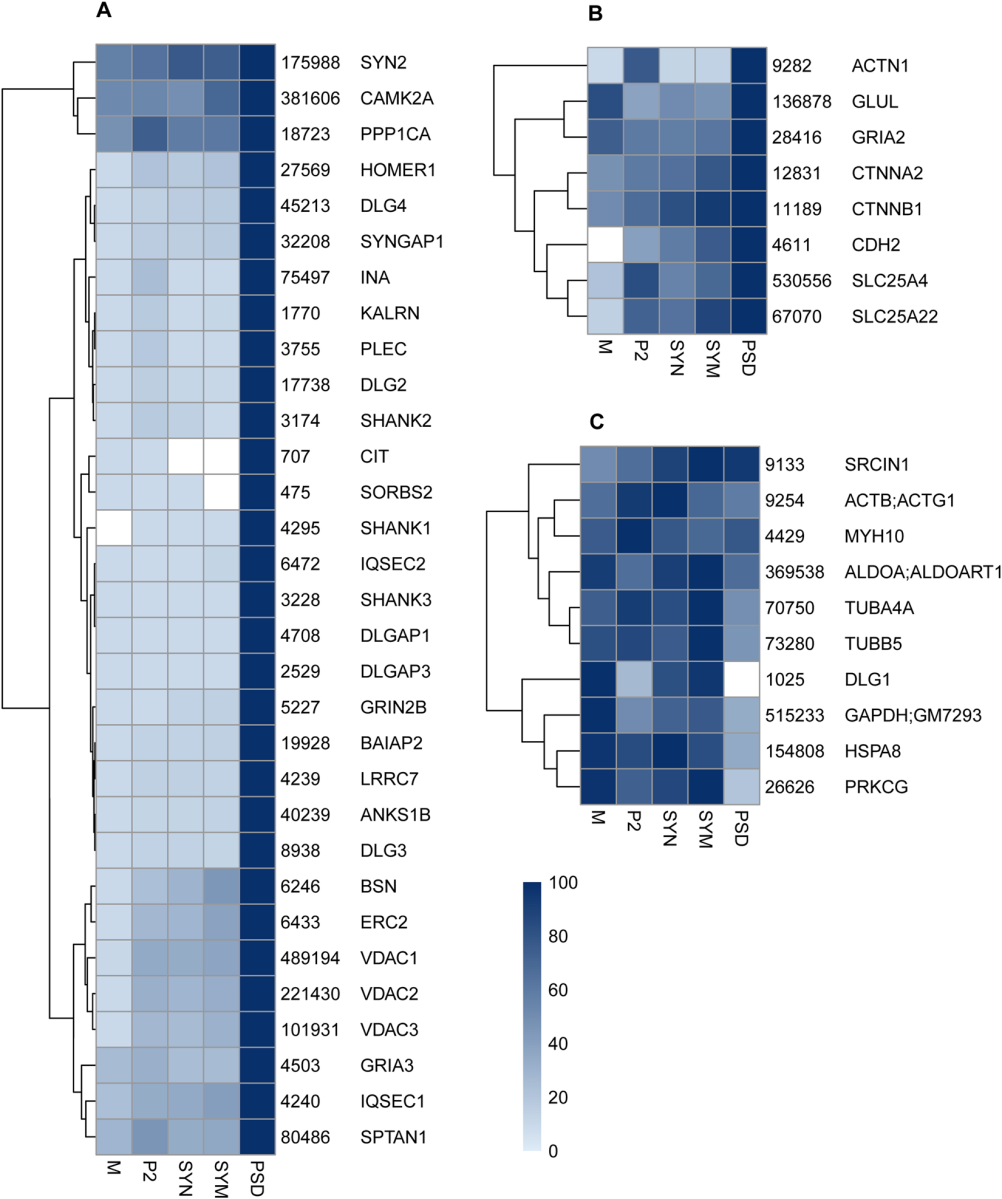
Comparison of the 49 PSD-95 (*DLG4*)-affinity associated proteins as listed in Table 2 from Dosemeci *et al*.^9^. (**A**) 31 proteins from this reference set are recovered by correlation analysis with hippocampal sub-fractions (as used for Fig. 4B). (**B**) 8 additional reference proteins are recovered using the Pearson correlation threshold from 0.9 to 0.5 (for 2 + reference proteins). (**C**) 10 remaining proteins from the reference set which were not recovered by correlation analysis. Summed iBAQ abundance of each protein is shown alongside each protein name. Protein abundances were scaled between zero and 100% of their maximum over all sub-fractions and color-coded from light-blue to dark-blue according to the heatmap.

Correlation profiling may indicate proteins that typically co-localize in a given subcellular localization. An alternative approach is to affinity isolate the organelle of interest for quantitative proteomics analysis. This might yield higher organelle purity than that obtained from biochemical isolation, but the presence of contaminants in an affinity-precipitated sample can still not be excluded^19,22^. To benchmark our approach, we compared the proteins from *GRIN2B-DLG4-HOMER1* set (cf. Fig. 4B) to the previously reported affinity purified PSD using an anti-DLG4 antibody (Fig. 5). Out of the top ranking proteins from the affinity isolated PSD (Table 2 from Dosemeci *et al*.^9^, containing 49 unique proteins), 31 were contained in the *GRIN2B-DLG4-HOMER1* set and 8 were enriched in PSD with lower correlation (between 0.5–0.9). Of the remaining 10 proteins, 1 showed a slightly weaker correlation of 0.49, 9 had no to negative correlation. The non-compliance of these proteins may be explained at least in part by their major localization site outside the PSD/spine. For example, tubulins (found in affinity-purified PSD) are abundantly present in the dendritic shaft, i.e. outside the synapse, for long distant transport and mechanical stability, whereas only a small fraction of microtubules may protrude into spine in an activity dependent manner^23^ and was reported to be present within PSD^24^. Thus, correlation profiling generates data that also includes information on enrichment and alternative distribution, whereas affinity isolation only addresses co-assembled proteins in the context of the bait.

Of particular interest, different protein family members can differ in their correlation profiles. For example, *DLG2, DLG3* and *DLG4* displayed the same PSD-enriched profile, different from *DLG1. DLG1* was reported involved in multiple functions, i.e. biosynthesis and trafficking of glutamate receptors^25,26^, as well as the recruitment of components of vesicle trafficking machinery either to the plasma membrane or to transport vesicles^27^. Also, *GRIA3* followed the *DLG4* profile, whereas *GRIA2* showed a lower correlation. The majority of *GRIA2* is present in GluA1/2 receptors that are known to have high membrane mobility. In fact, in hippocampus, there is a sizeable amount of extra-synaptic AMPAR, and AMPAR in synaptic cytosol and dendrites, which may form reserve or recycling pools for activity-dependent plastic changes of PSD-trapped AMPARs^28^. The latter may explain the observed *GRIA1* and *GRIA2* in the microsome fraction, resulting in a lower correlation to the *GRIN2B-DLG4-HOMER1* profile. Correlation profiling clearly reveals the house keeping proteins, such as *ALDOA* and *GAPDH* as not PSD specific (Fig. 5C).

### Glutamate receptors and their interacting proteins

**Figure 6:**
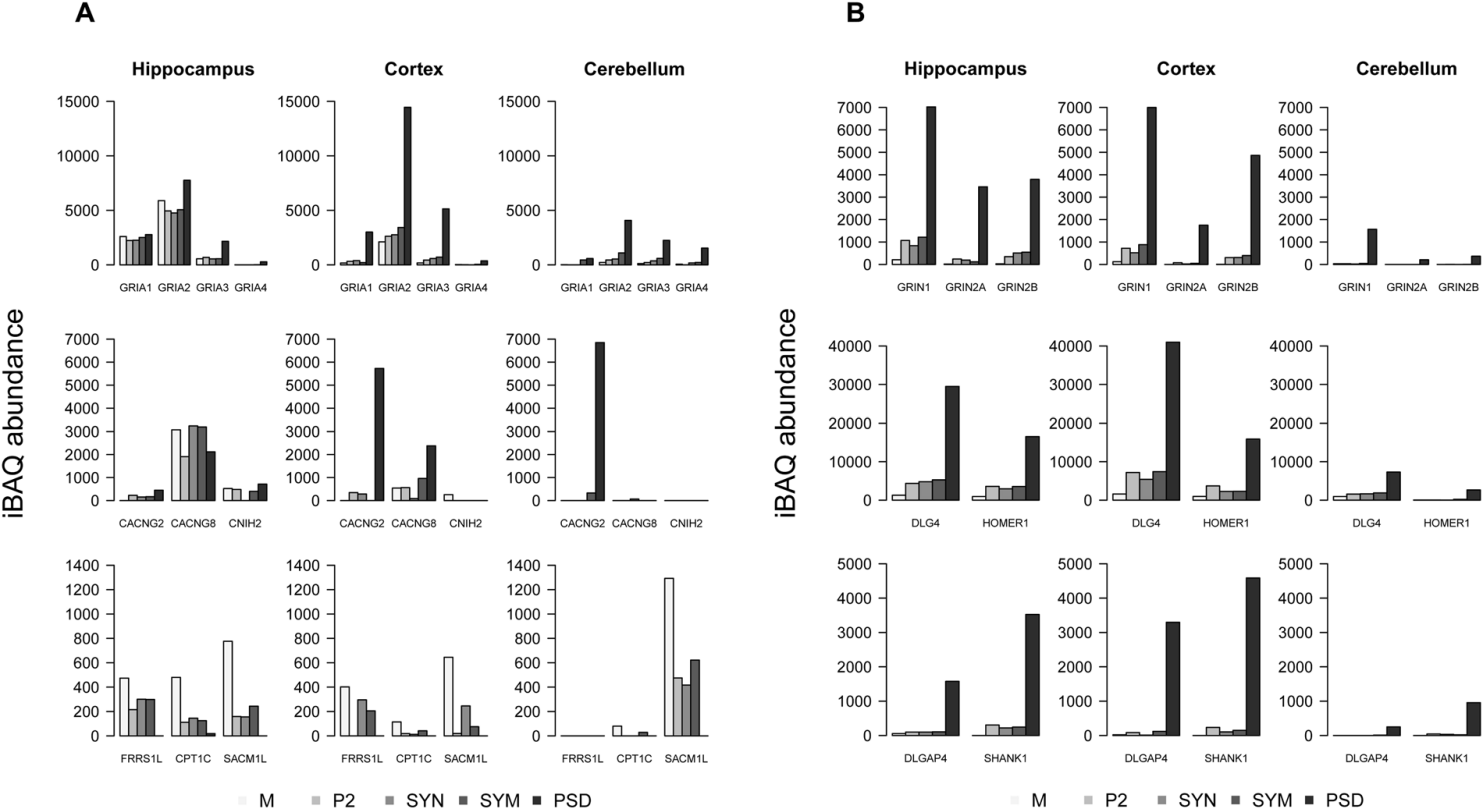
The sub-cellular abundance of ionotropic glutamate receptors and their associated proteins in three brain regions. (**A**) AMPA receptor subunits *GRIA*1-4, and 6 known auxiliary subunits, compared between sub-fractions and brain regions. Each group in the bar graph reflects the abundance of a protein over sub-fractions. (**B**) NMDA receptor subunits *GRIN1* and *GRIN 2 A/B*, and the interactors *DLG4, DLGAP4, HOMER1* and *SHANK1*. Legend at the bottom reflects the order of sub-fractions and their respective gray scale coding. Protein iBAQ values are computationally approximated absolute abundances.

Interestingly, the *GRIA* subunits forming AMPARs were highly enriched in cortex and cerebellum PSD and to a lesser extend in hippocampus. Compared to the *GRIN2B-DLG4-HOMER1* profile, *GRIA3* and *GRIA4* showed a 0.99 Pearson correlation (median value of these seed proteins) in all three brain regions whereas *GRIA1* and *GRIA2* showed a lower PSD enrichment in hippocampus reflected by 0.66 and 0.88 correlations, respectively. The percentage of *GRIA1* in the PSD fraction of cortex, cerebellum and hippocampus were 74%, 55% and 23%, respectively (Fig. 6A). This indicates a by far higher fraction of AMPARs anchoring to the PSD in cortex and cerebellum. Consequently, a lower percentage of AMPARs in cortex and cerebellum might be available for trafficking in and out of the PSD, which may impact on the extent of post-synaptic plasticity.

Equally, AMPAR interactors as previously established^18,19^ exhibited highly variable distribution patterns across the sub-fractions (Fig. 6A). For example, *CACNG2* was enriched in the PSD, *CPT1C* and *SACM1L* were highly enriched in microsome. In hippocampus and cortex, *FRRS1L* has a more even distribution in microsome and synaptosome but not in PSD. This is in general agreement with the previous studies indicating the co-localization of a population of AMPA receptors with *CPT1C* intracellularly rather than on the cell membrane^29^, whereas *CACNG2* traps AMPA receptors at the PSD and modulates channel properties^30,31^. Different members of the CACNG family showed pronounced brain-region specific expression differences. *CACNG2* is the predominant form of CACNG in the cerebellum residing mainly in the PSD. *CACNG8* is more abundant in hippocampus, and is widely distributed across all the examined sub-cellular compartments. This is in agreement with a recent study showing the restricted distribution of *CACNG2* mainly at the perforated synapses of pyramidal cells and the synapses of parvalbumin-positive interneurons, whereas *CANCG8* was present at various synapse types^32^.

Unlike the differential distribution of AMPAR interactors, the NMDAR subunits and their interactors *DLG4, DLGAP4, HOMER1* and *SHANK1* showed high enrichment in PSD in all three brain regions (Fig. 6B).

### Postsynaptic density proteins

**Figure 7:**
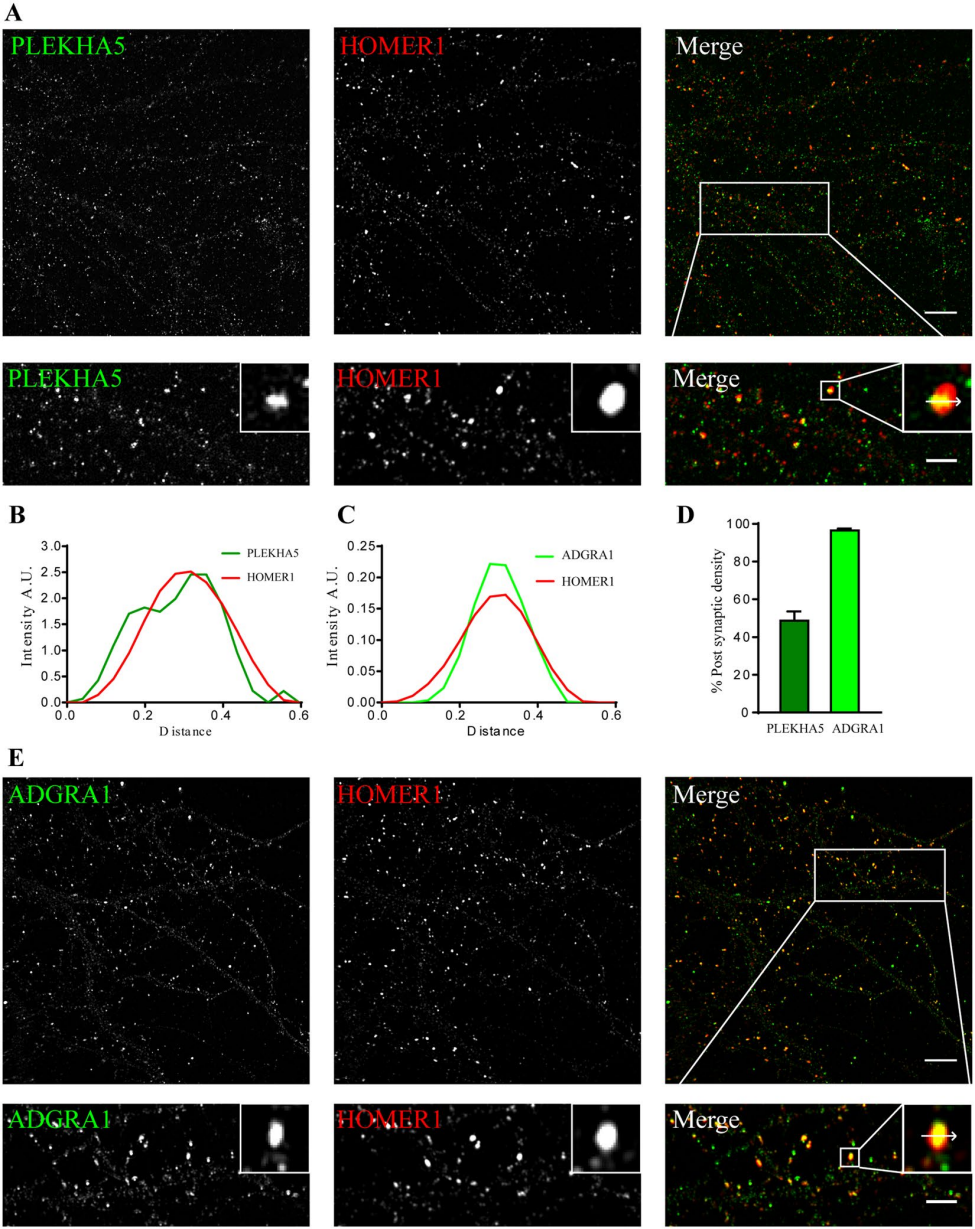
Super-resolution imaging microscopy validation of novel PSD-enriched proteins, *PLEKHA5* and *ADGRA1*. (**A**,**E**) SIM imaging of primary cultured hippocampal neurons at DIV19 for *PLEKHA5* and *ADGRA1* (green) along with *HOMER1* (red), the latter as a marker for the PSD. Lower panel shows zoom in of the marked area (scale bar 2 µm). (**B**,**C**) Line scan analysis on inset from panel a,e shows co-localization between the three proteins at postsynaptic sites. (**D**) Bar graph showing percentage (mean ± sem) of postsynaptic density *HOMER1* puncta positive for *PLEKHA5* (dark green, 48.61 ± 4.92, n = 10), *ADGRA1* (light green 96.54 ± 1.02, n = 7).

Proteins that share similar profile have a high chance to be present in the same subcellular compartment. We selected two PSD proteins for further study, namely *ADGRA1* and *PLEKHA5*, which showed high correlation to the *GRIN2B-DLG4-HOMER1* profile and were found in the PSD fraction of all three brain regions. *PLEKHA5* was found previously in a PSD preparation^33^, but PSD localization has never been demonstrated. Here, we performed super-resolution structured illumination microscopy and showed that *PLEKHA5* co-localizes with the PSD marker Homer (Fig. 7A,B), thereby confirming it as a PSD protein. *PLEKHA5* was present in about 50% of synapses (Fig. 7D). In contrast, *ADGRA1* was exclusively found in the PSD fraction with an iBAQ value 300 fold lower than *GRIA1*, showing that its copy number in the PSD is very low, and it might have been missed in previous experiments because of this. We observed excellent overlap of *ADGRA1* and *HOMER1* in primary hippocampal cultures (Fig. 7E). Line scan analysis of synapses showed nearly perfect co-localization of *HOMER1* and *ADGRA1* (Fig. 7C). We further quantified the percentage of *ADGRA1* positive synapses and revealed that 96.54% of *HOMER1* positive puncta representing synapses were positive for *ADGRA1* (Fig. 7D).

*ADGRA1* is present on chromosome 10q26.3 and is a 7-TM domain^34^ containing protein belonging to the adhesion family of G protein-coupled receptors. It is predicted to have a PDZ binding domain in the C-terminus of the protein^35^. It is the only member of the adhesion GPCRs that lacks a cleavable GPCR auto-proteolysis–inducing (GAIN) domain^36^. Due to its widespread expression in the brain, it is suggested that *ADGRA1* may have an important role in the regulation of neuronal signal transduction. It has been reported in complex with PSD95 (*Dlg4*)^37^, which is in line with its tight localization within the PSD. Further research on the functions of *ADGRA1* might elucidate the processes in which it is involved.

*PLEKHA5* is a cytosolic protein belonging to the *PLEKHA5* family and is a Pleckstrin Homology (PH) Domain containing protein through which it is involved in binding to Phosphatidylinositol (3, 4, 5)-trisphosphate (PIP_3_)^38^. Previous PSD identification studies from human postsynaptic densities have identified *PLEKHA5* in the postsynaptic density preparations, but validation of its PSD localization was not reported^33^. Interestingly, *PLEKHA5* belongs to region 12p12 and SNPs associated with this locus are associated with early onset bipolar disorder^39^. Like other PH domain containing proteins, *PLEKHA5* might get recruited to the plasma membrane upon PIP_3_ formation in the postsynaptic compartments. The role that it plays in the postsynaptic density remains to be determined.

In conclusion, correlation profiling of synaptic sub-fractions aids to reveals interactome organization and may help to define subcellular structures of interest^17,40^. Typically, immunoblotting of synaptic protein distribution corresponded well with our sub-fraction proteomics data. When comparing our data with a previously reported immuno-purified PSD protein complex, we observed high degree of agreement. Significantly, our large dataset with thousands of proteins covering different synapse sub-fractions from three brain regions allows to interrogate the biochemically-defined spatial distribution of most synaptic proteins in a brain-region specific manner, and may help to generate hypotheses regarding novel protein localizations related to synapse functions.

## Materials and Methods

### Preparation of cellular sub-fractions

All animal experiments were performed in accordance with relevant guidelines and regulations of the VU University. The animal ethics committee of the VU University approved the experiments. Both male and female mice were used.

Subcellular fractions were prepared from 3-month-old C57BL6 mice as described in^41^. In brief, mouse hippocampi, cortex and cerebellum were dissected and stored at −80 °C until used. In total 45 mice were used for 3 hippocampus replicates, 4 mice for cortex, and 30 mice for cerebellum. The brain regions were pottered separately in homogenization buffer (0.32 M Sucrose, 5 mM HEPES pH 7.4, Protease inhibitor cocktail (Roche)) on a dounce homogenizer (potterS; 12 strokes, 900 rpm) and spun at 1000 × g for 10 min at 4 °C. Supernatant 1 (S1) was centrifuged at 20,000 × g for 20 min to obtain pellet 2 (P2) and supernatant 2 (S2). The S2 fraction was ultracentrifuged at 100,000 × g for 2 hrs; the pellet was recovered as microsomal fraction. S1 was subjected to ultracentrifugation in a 0.85/1.2 M sucrose density gradient at 100,000 × g for 2 hrs. Synaptosomes were recovered at the interface of 0.85/1.2 M sucrose. The hypotonic shock of synaptosomes in 5 mM HEPES with protease inhibitor for 15 min yielded the synaptic membrane fraction, which was subsequently isolated by sucrose gradient ultracentrifugation as stated above at the interface of 0.85/1.2 M fraction. To obtain the PSD, the synaptosome fraction was extracted in 1% Tx-100 for 30 min, layered on top of 1.2/1.5/2 M sucrose, centrifuged at 100,000 × g for 2 hrs, and recovered as PSD-I at the interface of 1.5/2 M sucrose. PSD-I was subjected to second extraction in 2% Tx-100 for 30 min, subjected to sucrose gradient ultracentrifugation as stated above, and recovered at the 1.5/2 M sucrose interface. The PSD-II fraction was then pelleted in 5 mM HEPES by centrifuging at 100,000 × g for 30 min.

### Gel separation and in-gel digestion

Gel digestion was performed as described^22,42^. Sample was dissolved in Laemmli buffer and boiled at 98 °C for 5 min; 5 µl 30% acrylamide was then added and vortexed for 30 min at room temperature to form a fixed modification of Cys-S-beta-propionamide. Samples were run on a 10% SDS-PAGE gel, which was stopped when the front reached halfway of the gel. The gel was fixed overnight in 40% ethanol/3% phosphoric acid, and stained briefly for about 30 min with colloidal coomassie blue. Each lane was cut into two slices, chopped into 1 mm by 1 mm pieces followed by a sequential incubation in 50% acetonitrile/50 mM NH_3_HCO_3_ −100% acetronitrile −50 mM NH_3_ HCO_3_ −50% acetonitrile/50 mM NH_3_HCO_3_ −100% acetronitrile. The gel pieces were dried in a speedvac, rehydrated in trypsin solution in 50 mM NH_3_HCO_3_ (500 ng per gel slice) at 37 °C overnight, extracted with 200 µL 0.1 M acetic acid, and the supernatant was transferred to an Eppendorf tube and dried in a speedvac. The tryptic peptides were dissolved in 17 µL 0.1 M acetic acid and analyzed by LC-MS/MS.

### MS acquisition and data analysis

Peptides were analyzed by nano-LC MS/MS using an Ultimate 3000 LC system (Dionex, Thermo Scientific) coupled to the TripleTOF 5600 mass spectrometer (Sciex)^43^. Peptides were trapped on a 5 mm Pepmap 100 C18 column (300 μm i.d., 5 μm particlesize, from Dionex) and fractionated on a 200 mm Alltima C18 column (100 μm i.d., 3 μm particle size). The acetonitrile concentration in the mobile phase was increased from 5 to 30% in 90 min, to 40% in 5 min, and to 90% in another 5 min, at a flow rate of 500 nL/min. The eluted peptides were electro-sprayed into the TripleTOF MS. The nano-spray needle voltage was set to 2500 V. The mass spectrometer was operated in a data-dependent mode with a single MS full scan (m/z 350–1200, 250 msec) followed by a top 25 MS/MS (85 msec per MS/MS, precursor ion >90 counts/s, charge state from +2 to +5) with an exclusion time of 16 sec once the peptide was fragmented. Ions were fragmented in the collision cell using rolling collision energy, and a spread energy of 10 eV.

The MS raw data were imported into MaxQuant (version 1.5.2.8)^44^, and searched against the UniProt mouse proteome (SwissProt + Trembl February 2016 release) with Cys-S-beta-propionamide as the fixed modification and Methionine oxidation and N-terminal acetylation as variable modifications. For both peptide and protein identification a false discovery rate of 0.01 was set, MaxLFQ normalisation was enabled with a LFQ minimal ratio count of 1. The minimal peptide length was set to 6; further MaxQuant settings were left at default. The MaxQuant search results are provided in Supplementary Table S6. The mass spectrometry proteomics data have been deposited to the ProteomeXchange Consortium via the PRIDE^45^ partner repository with the dataset identifier PXD005634.

External contaminants such as immunoglobulins, keratin and trypsin, as well as histones, were excluded from downstream analysis.

Next, we collapsed protein groups that shared the same gene name. All (majority) protein accessions in a protein group were matched against the Fasta database, their gene names extracted from the Fasta headers and the set of unique gene names for each protein group was stored. The protein abundance matrix was built by summation of MaxLFQ normalised protein intensities of protein groups that map to the same unique set of genes. These protein intensities were converted to iBAQ pseudo-absolute abundances using the number of digestible peptides provided by MaxQuant and then replicates were merged using their respective mean protein abundances (missing values were disregarded). This data is provided in Supplemental Table S1.

### Immunostaining of primary neurons

Primary hippocampal neurons were obtained from E18 rat pups as described previously^46^. Briefly, 18000 cells were grown in neurobasal medium supplemented with B27 on poly D-Lysine coated coverslips. The cells were used for staining at DIV 14–16. The coverslips were fixed with ice-cold methanol for 10 min, followed by three washes in ddH_2_O and PBS. The neurons were then blocked and permeablised with blocking buffer (5% FCS, 0.1% Triton X-100, and 0.1% Glycine in phosphate buffered saline, pH 7.4) for 1 hour. Next, the neurons were incubated with anti-*ADGRA1* (GPR123) (1 in 250, cat. no. sc-162892, Santa cruz) or anti-*PLEKHA5* (1 in 250, cat. no. sc-390311, Santa cruz) and anti-*HOMER1* (1 in 1000, cat. no. 160 004, Synaptic systems), diluted in blocking buffer overnight at 4 °C. After three times washing in PBS, the cells were incubated with an alexa conjugated secondary antibodies for 1 hour at room temperature (anti-mouse Alexa 488 (1 in 1000), anti-goat Alexa 488 (1 in 1000), Anti-Guinea pig Alexa 647 (1 in 1000) (Molecular Probes) and subsequently washed and fixed on glass slides (Superfrost Plus, Thermo) using Moviol. Images were taken using a LSM Elyra SIM microscope with 63x Oil immersion lens (N.A. 1.4) and analyzed using ImageJ. Line scan analysis was performed as described in^46^.

### 3D-SIM microscopy

Imaging was performed using a Zeiss Elyra PS1 system. 3D-SIM data was acquired using a 63 × 1.4NA oil objective. 488 nm, 561 nm, 642 nm, 100 mW diode lasers were used to excite the fluorophores together with respectively a BP 495–575 + LP 750, BP 570–650 + LP 750 or LP 655 emission filter. For 3D-SIM imaging a grating was present in the light path. The grating was modulated in 5 phases and 5 rotations, and multiple z-slices were recorded with an interval of 110 nm on an Andor iXon DU 885, 1002 × 1004 EMCCD camera. Raw images were reconstructed using the Zeiss Zen software.

Reconstructed 3D-SIM images were analyzed with imageJ^47^ extended in the FIJI framework^48^. Particles larger than 10 pixels were detected and marked as region of interest (ROI) and mean *ADGRA1* or *PLEKHA5* signals inside the ROIs were measured. A *HOMER1* particle was counted as positive for *ADGRA1* or *PLEKHA5* if their mean intensity was more than three times above their respective local backgrounds.

### Data Availability

The datasets generated during the current study are available in the the ProteomeXchange Consortium via the PRIDE partner repository with the dataset identifier PXD005634.

## Electronic supplementary material

Supplementary Figure S1-3

sTable1

sTable 2

sTable 3

sTable 4

sTable 5

sTable 6
