# Supplementary material for: Correlation profiling of brain sub-cellular proteomes reveals co-assembly of synaptic proteins and subcellular distribution
Source: Sci Rep. 2017 Sep 21;7:12107. doi: 10.1038/s41598-017-11690-3 (PMC5608747; doi:10.1038/s41598-017-11690-3)
Supplement: Supplementary file 1 — Supplementary Figure S1-3 [file 41598_2017_11690_MOESM1_ESM.pdf]

Correlation profiling of brain sub-cellular proteomes reveals co-assembly of synaptic proteins and subcellular distribution

Nikhil J. Pandya, Frank Koopmans, Johan A. Slotman, Iryna Paliukhovich, Adriaan B. Houtsmuller, August B. Smit, Ka Wan Li

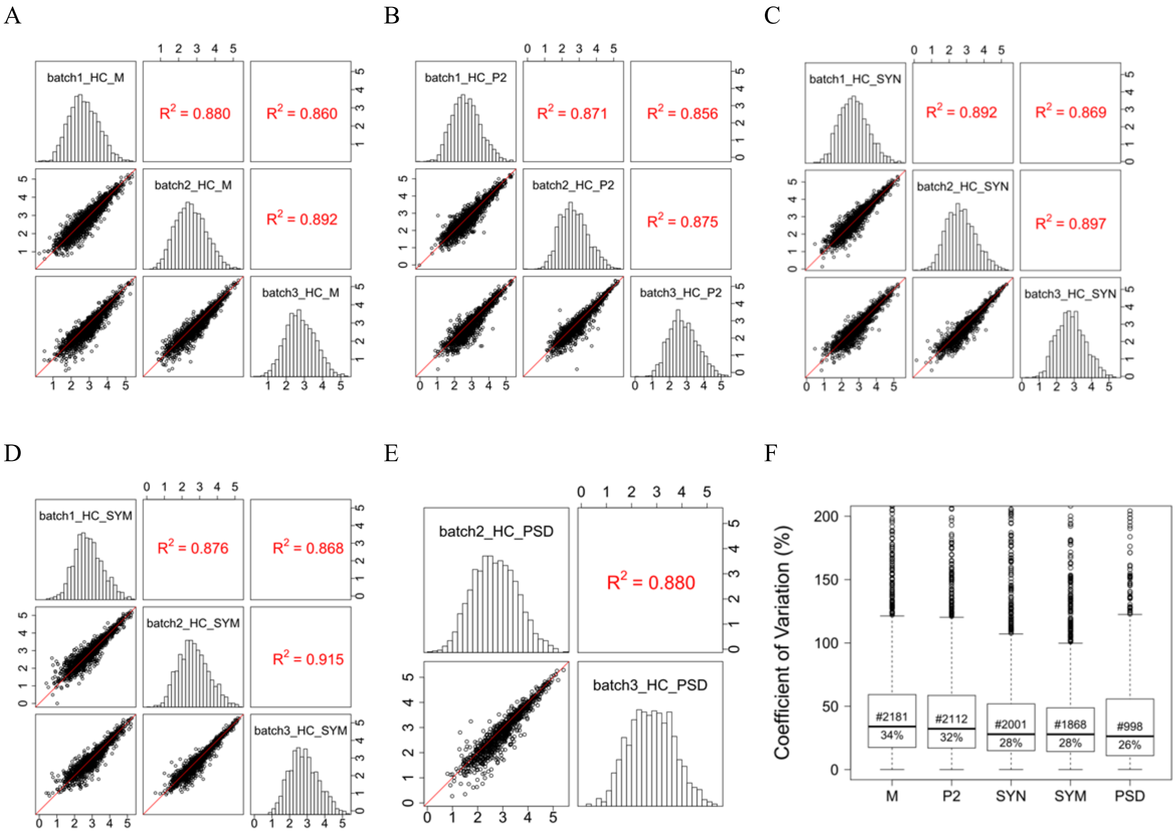

Supplementary Figure S1. Reproducibility of subcellular fractions. (A-E) A pairwise analysis of replicates, each panel represents a hippocampal sub-fraction. Histograms representing the protein abundance distribution of individual samples are shown on the diagonal. The pairwise reproducibility of samples is illustrated by scatterplots and quantified by their respective coefficient of determination (R^2). For the PSD we obtained 2 instead of 3 replicates, which were highly similar in accordance to the reproducibility of other sub-fractions. Protein abundances were log10 scaled (x-axis for histograms, x- and y-axis for scatterplots). (F) Coefficient of Variation (CoV) between replicates of each sub-fraction. Numbers inside boxes indicate the number of proteins quantified in at least 2 replicates and the median CoV of those proteins.

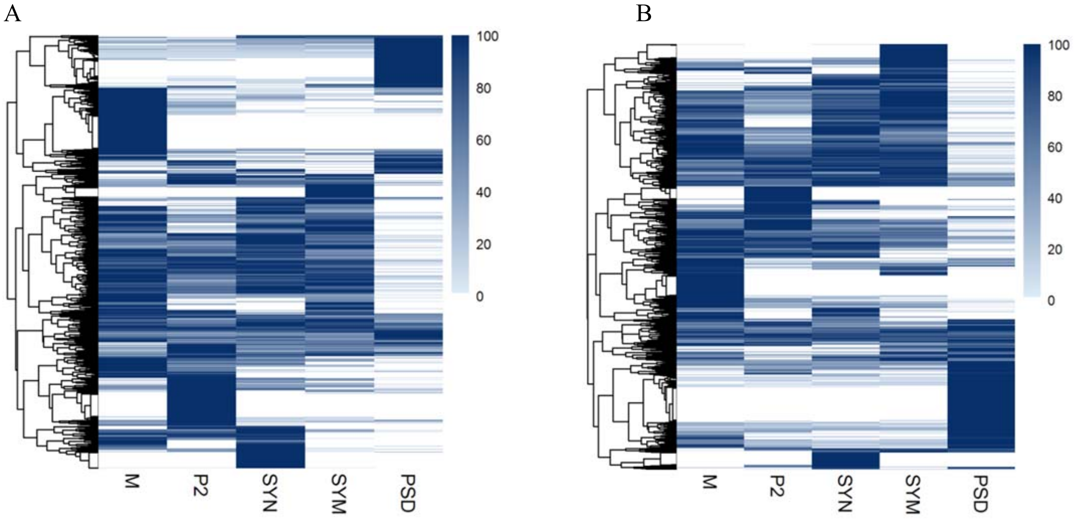

Supplementary Figure S2. Related to Figure 3. Hierarchical clustering of 2912 and 3311 proteins in various subcellular fractions of the A) cortex and B) cerebellum, respectively. Protein abundances were scaled between zero and 100% of their maximum over all sub-fractions.

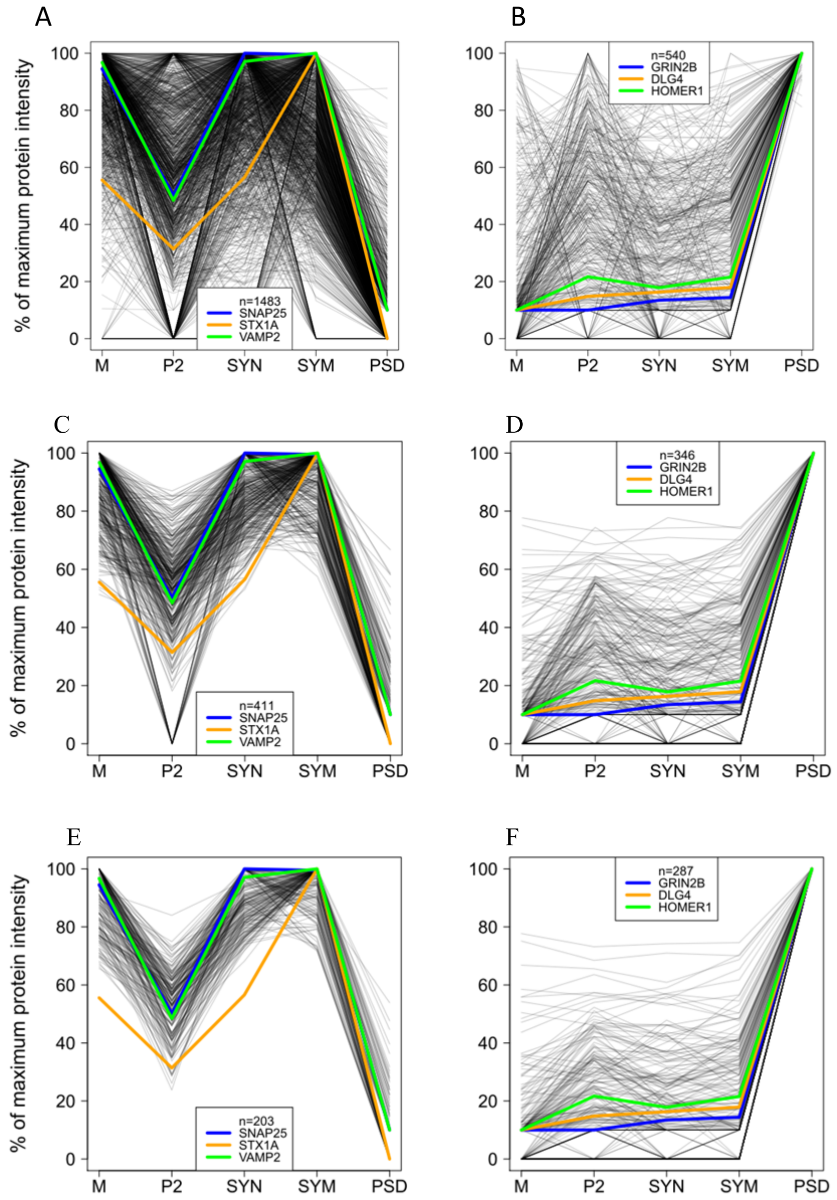

Supplementary Figure S3. Related to Figure 4. Correlation analysis of abundance profiles of proteins over sub-fractions of the hippocampus with selected seed proteins. Three functionally related seed proteins were chosen typical for exocytosis (*SNAP25, STX1A, VAMP2)* or *PSD (GRIN2B, DLG4, HOMER1*). Panel A, B, Pearson correlation of ≥0.5 with at least two out of the three seed proteins. Panel C, D, Pearson correlation of ≥0.9 with at least two out of the three seed proteins. Panel E, F, Pearson correlation of ≥0.95 with at least two out of the three seed proteins. The resulting number of proteins is given in each figure. Protein abundances are scaled between zero and their maximum intensity over all selected sub-fractions**.**
